# Catalysts for the Conversion of CO_2_ to Low Molecular Weight Olefins—A Review

**DOI:** 10.3390/ma14226952

**Published:** 2021-11-17

**Authors:** Barbara Pawelec, Rut Guil-López, Noelia Mota, Jose Luis Garcia Fierro, Rufino Manuel Navarro Yerga

**Affiliations:** Instituto de Catálisis y Petroleoquímica, Spanish National Research Council (CSIC), 28049 Madrid, Spain; rut.guil@icp.csic.es (R.G.-L.); noelia.mota@icp.csic.es (N.M.)

**Keywords:** CO_2_ hydrogenation, light olefins, tandem catalysts, direct olefin production

## Abstract

There is a large worldwide demand for light olefins (C_2_^=^–C_4_^=^), which are needed for the production of high value-added chemicals and plastics. Light olefins can be produced by petroleum processing, direct/indirect conversion of synthesis gas (CO + H_2_) and hydrogenation of CO_2_. Among these methods, catalytic hydrogenation of CO_2_ is the most recently studied because it could contribute to alleviating CO_2_ emissions into the atmosphere. However, due to thermodynamic reasons, the design of catalysts for the selective production of light olefins from CO_2_ presents different challenges. In this regard, the recent progress in the synthesis of nanomaterials with well-controlled morphologies and active phase dispersion has opened new perspectives for the production of light olefins. In this review, recent advances in catalyst design are presented, with emphasis on catalysts operating through the modified Fischer–Tropsch pathway. The advantages and disadvantages of olefin production from CO_2_ via CO or methanol-mediated reaction routes were analyzed, as well as the prospects for the design of a single catalyst for direct olefin production. Conclusions were drawn on the prospect of a new catalyst design for the production of light olefins from CO_2_.

## 1. Introduction

Light olefins (C_2_^=^–C_4_^=^) can be produced by steam cracking, fluid catalytic cracking of naphtha, direct/indirect conversion of synthesis gas (CO + H_2_) [1] or by hydrogenation of CO_2_ using H_2_ from renewable energy sources [2,3,4]. In particular, the latter method is very interesting for the research community and the chemical industry because it could mitigate global climate changes caused by the progressive increase in CO_2_ emissions to the atmosphere [5,6]. In fact, the use of light olefins as C-building blocks to produce a variety of chemicals (elastomers, medicines, cosmetics, detergents, solvents, etc.) could avoid up to 23% of CO_2_ emissions [5]. However, the formation of light olefins via CO_2_ hydrogenation reaction is difficult due to the chemical inactivity of the CO_2_ molecule, the high C–C coupling barrier and the necessity to limit the formation of C–C bonds and methane [7,8]. Therefore, the catalysts required for the production of light olefins must be multifunctional and have an optimized amount of active sites. This could be achieved with catalysts with a moderate hydrogenation function and active sites controlled by the addition of promoters. However, the addition of promoters alone cannot increase selectivity to levels of interest for the industry. In this regard, multifunctional metal–zeolite catalysts showing enhanced CO_2_ activation and inhibition of secondary olefin hydrogenation were intensively investigated [3].

Although the reaction of the CO_2_ hydrogenation to olefins is thermodynamically favorable, there are only a few very efficient catalytic systems exhibiting high selectivity to olefins. The main reason is that the catalyst has to be able to simultaneously catalyze the reverse water-gas shift (RWGS) and modified Fischer-Tropsch to olefins (FTO) reactions working under the same operating conditions, and its final behavior depends on several combined factors, such as the type of metals, the selection of supports, the co-catalyst and the method of catalyst preparation and the selection of operating conditions. For catalyst design, the selection of metals is limited to Fe, Co, Pd, Ni and Ru, which are the only ones that show high activity in the CO_2_ hydrogenation upon moderate reaction conditions. Among them, only Fe-based catalysts show relatively high selectivity towards desired products while maintaining low selectivity towards undesired methane [9]. In addition, Fe-based catalysts are attractive to the industry due to their low cost, which is related to the abundance of iron in nature, and flexible product distribution [10,11].

Since light olefins are in high demand, there is a large body of work related to their production by CO_2_ hydrogenation ([7,8,9,10,11,12,13,14,15,16,17,18] and references within). Recently, several published reviews focused on different aspects of catalyst design for the improvement of olefins production through this reaction [3,4,12,13,14,15,16,17,18]. For example, the utilization of composite catalysts or multifunctional catalysts for this reaction was reviewed by Guo et al. [13], while Zhou et al. [14] reviewed the breaking of the selectivity limitation in the transformation of syngas and CO_2_ hydrogenation into chemical hydrocarbons and fuels. More recently, Ma and Porosoff [3] and Numpilai et al. [18] reviewed advances in the development of tandem catalysts. Since these reviews, a large number of new papers have appeared on the production of olefins from synthesis gas and CO_2_. Their constant review is necessary to avoid the repetition of unnecessary work and to critically evaluate the practical feasibility of new catalyst formulations.

We dedicate this work to the recently deceased Professor José Luis García Fierro, whose research group investigated the catalytic aspects of the Fischer–Tropsch synthesis process for more than two decades [1,9,19,20,21,22,23,24,25,26].

## 2. Reaction Mechanism

**Figure 1:**
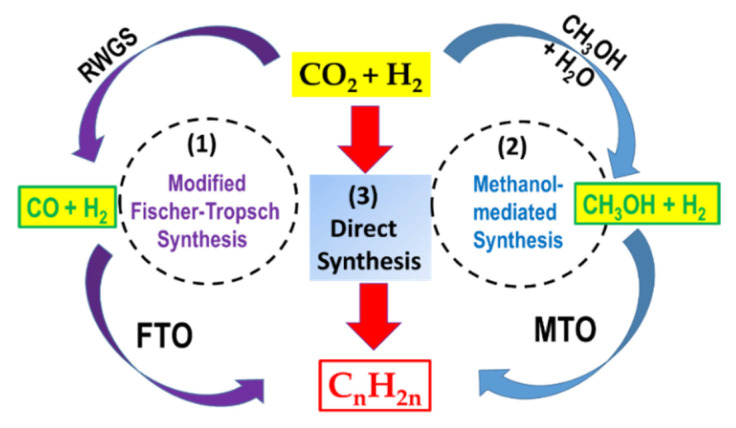
General scheme of the three routes of olefins formation from CO_2_: (1) modified Fischer–Tropsch to olefins (FTO) route; (2) methanol to olefins (MTO) route; (3) direct olefin formation over multifunctional, promoted catalysts.

Depending on the catalyst used, the formation of olefins from CO_2_ can occur via various alternative routes (Figure 1): (1) CO-mediated modified Fischer–Tropsch to olefins (FTO) route; (2) methanol to olefins (MTO) route [27]; (3) direct CO_2_ hydrogenation to olefins using promoted and multifunctional catalysts (Equation (1)). The latter reaction pathway is kinetically more difficult and is still under development. In particular, the role of the promoter to enhance CO_2_ conversion and selectivity towards olefins formation need to be investigated [3].

nCO_2_ + 3nH_2_ → C_n_H_2n_ + 2nH_2_O      Δ*H*^0^_573K_ = 128 kJ mol^−1^ (1)

In general, it is observed that the FTO route (1) is more efficient in terms of hydrocarbon yield than the MTO route.

### 2.1. Modified Fischer–Tropsch to Olefins (FTO)

The production of olefins from CO_2_ through the modified Fischer–Tropsch synthesis pathway occurs by two different reaction mechanisms:

(1) Redox mechanism: CO is formed by adsorption and activation of CO_2_ on metals or metal oxides, and H_2_ does not participate in the formation of intermediate products (Equation (2));

CO_2_ + H_2_ → CO + H_2_O     RWGS reaction     Δ*H*^0^_298_ = 42.1 kJ mol^−1^ (2)

(2) Associative mechanism: multi-site adsorption of reagents, chain initiation, chain growth and termination and desorption of the final product (Equation (3)). In this mechanism, the association of hydrogen with CO_2_ leads to the formation of different intermediate surface species, such as formate, carbonate or carboxylic species, which decompose into final products [26].

CO + 2H_2_ → (-CH_2_-) + H_2_O → C_n_H_2n_ + H_2_O     FTO reaction     Δ*H*^0^_298_ = −152.0 kJ mol^−1^ (3)

The RWGS is an endothermic reaction, so it is favored at higher temperatures, whereas FTO is an exothermic process [28,29]. Therefore, thermodynamic data indicate that low temperature favors the FTO reaction, while a high temperature is necessary to activate CO_2_ for the RWGS reaction [3,4]. Therefore, the reaction conditions greatly influence the CO_2_ conversion and product distribution. In this regard, it is important to note that a low temperature required for the FTO reaction also favors the exothermic methane synthesis reaction (Equation (4)):

CO_2_ + 4H_2_ → CH_4_ + H_2_O     methane synthesis     Δ*H*^0^_298_ = −165 kJ mol^−1^ (4)

Although the mechanism of the CO_2_ hydrogenation reaction has been extensively investigated for a long time, it has not yet been fully understood. This is probably because a wide variety of O-, H- and C-containing species are present on the catalyst surface, and that all of them may be involved in the reaction mechanism mediated by the CO. The main challenges of Fe-based FTO catalysts are the increase in selectivity towards light olefins, the decrease in methane production and the reduction in excessive CO_2_ formation. To maximize olefin production in all reaction routes, the formation of side products, such as CO, CH_4_, C_2_–C_4_ alkanes and C_5+_ hydrocarbons, should be avoided.

**Figure 2:**
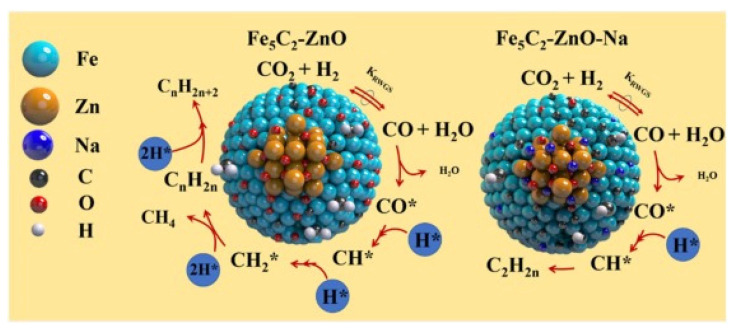
Reaction mechanism of CO_2_ hydrogenation over Na-free and Na-doped Fe_5_C_2_–ZnO catalysts. Reproduced from Ref. [32] with copyright license from Elsevier (2021).

There are a large number of theories and hypotheses presented in the literature on the possible mechanism of the modified FT reaction. This is due to the structure sensitivity of CO_2_ activation [30] and the dependence of CO_2_ hydrogenation on CO_2_ coverage and hydrogen adsorption mode [31]. Therefore, the reaction mechanism depends on the catalyst formulation, catalyst morphology, catalyst pretreatment, reaction conditions employed and proper hydrogen dosing according to the reactor configuration [12]. As an example, Figure 2 shows the reaction mechanism recently proposed for Na-free and Na-doped Fe_5_C_2_–ZnO catalysts by Tu et al. [32]. Considering the higher surface ratio of CH* to CH_2_ in the Na-modified catalyst, it was proposed that the formation of surface CH–CH* species, relevant for olefin formation, occurs through the C–C bond coupling of two CH* species on the Fe(100) surface [32].

In general, the modified FT route of CO_2_ hydrogenation could occur via redox (dissociation of CO_2_ to CO and O) or via an associative mechanism, in which hydrogen reacts with CO_2_ to form the intermediate HOCO. This intermediate then decomposes into CO and OH, the latter being hydrogenated to H_2_O. In addition, there is also evidence for the third reaction mechanism leading to the formation of stable HCOO species [33]. For example, by applying the Born–Oppenheimer molecular dynamics simulation, Lin et al. concluded that, even for low kinetic energies, both Eley–Rideal and hot atom mechanisms occur [31].

Unfortunately, in the FTO reaction, product selectivity depends on chain growth, which can be predicted using the chain growth probability (α) of the Anderson–Schulz–Flory (ASF) distribution model, which can be calculated by the following equation (5):

W_n_ = n(1 − α)^2^ α^n−1^ (5)

where W_n_ is the mass fraction of hydrocarbons with carbon number n in the chain and α is the chain growth probability, which is a function of the rates of chain growth and chain termination [34].

Different factors are known to influence the α-chain growth parameter, such as the process conditions, the nature of the catalyst and the presence of some additives or catalyst promoters. According to the Anderson–Schulz–Flory model, the maximum selectivity towards C_2_–C_4_ hydrocarbon fraction is about 56.7%, while maximum selectivity toward undesired methane is about 29.2% [35]. As can be deduced from the ASF product distribution, the chain growth parameter α below values of 0.3 could result in excessive methane production. This limiting value was considered a major barrier for the industrial application of direct conversion of CO_2_ + H_2_ gas mixture into small olefins via the FTS route [36]. As a consequence of the ASF limitation, the olefin selectivity of FTO catalysts is generally less than 60% [37].

The simplest way to maximize olefin production is to operate at a high temperature when the RWGS reaction (Equation (2)) is produced together with the FTO reaction (Equation (3)). In fact, the CO_2_ hydrogenation is typically studied at temperatures higher than 300 °C. In the case of Fe-based catalysts, the formation of oxygenates and hydrocarbons is believed to occur through the CO insertion and surface carbide mechanisms, respectively. In the case of the CoCu/TiO_2_ catalyst, the recent study by Shi et al. [38] suggests that the CO_2_ molecule can be initially reduced to CO by H_2_ via RWGS at the Cu sites, followed by hydrogenation of CO to olefins via the FTO reaction at the Co sites.

### 2.2. Methanol-Mediated Olefin Synthesis (MTO)

As compared with the RWGS-FTS synthesis route, the high selectivity toward light olefins (up to 80%) can be achieved only at much lower CO_2_ conversion (about 13%). Higher CO_2_ conversions lead to lowering of olefins selectivity due to side reactions [39]. In order to overcome such a limitation, the methanol-to-olefins (MTO) process needs a bifunctional catalyst. The formation of light olefins occurs via the following reactions pathways:

CO_2_ + H_2_ → CH_3_OH + H_2_O      Δ*H*^0^_298_ = −45.9 kJ mol^−1^ (6)

CH_3_OH + H_2_ → C_n_H_2n_ + H_2_O      MTO (7)

**Figure 3:**
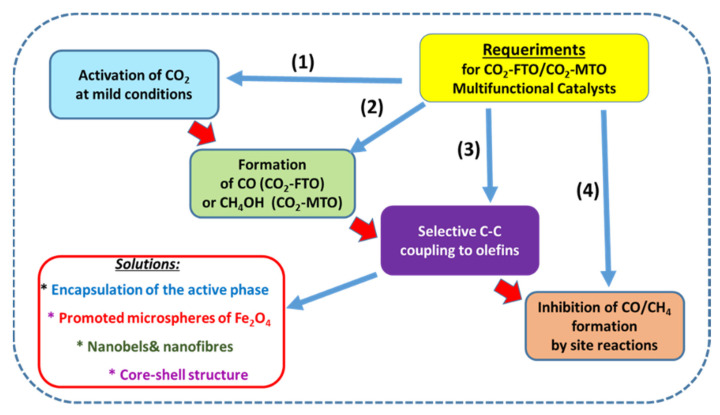
Visualization of the requirements for an ideal multifunctional catalyst for the production of olefins from CO_2_ via the FTO and MTO routes.

For the methanol-mediated route, the formation of side products is favored at high CO_2_ conversions. Therefore, to maximize olefin production, the formation of side products such as CO, CH_4_, C_2_–C_4_ alkanes and C_5_+ hydrocarbons must be avoided. Usually, the MTO route of olefins formation requires two separate reactors. In the first reactor, the synthesis of methanol occurs, whereas the synthesis of olefins from methanol over the zeolite catalyst component takes place in the second reactor. For the reaction carried out in the same reactor, the bifunctional catalyst with both metallic and acidic functions is needed [3,40]. The necessary requirements for olefin production over an ideal multifunctional catalyst are visualized in Figure 3. As can be seen, regardless of the reaction route, the ideal multifunctional catalyst can be capable of activating CO_2_ under mild conditions to form CO or CH_3_OH and then selectively transform those intermediates into olefins while avoiding site reactions which would lead to the formation of undesired products (CO, CH_4_, etc.).

## 3. Dynamic Character of the Catalytically Active Iron Phases

The nature of the active phase of iron catalysts are still under debate due to the dynamic character of the active phases during CO_2_ hydrogenation, originating from the complex interactions between the gaseous species (CO_2_, CO, H_2_) and the iron phases [41]. It is well known that the activity, selectivity and/or stability of the catalyst depend on the type of active phases, the formation of which depends on the process conditions, the activation procedure and the catalyst composition. 

Moreover, the relative concentration of these phases evolves during the reaction. The most active Fe phase in FTS has not been unequivocally identified yet because of the absence of effective in situ characterization techniques [3,18].

**Figure 4:**
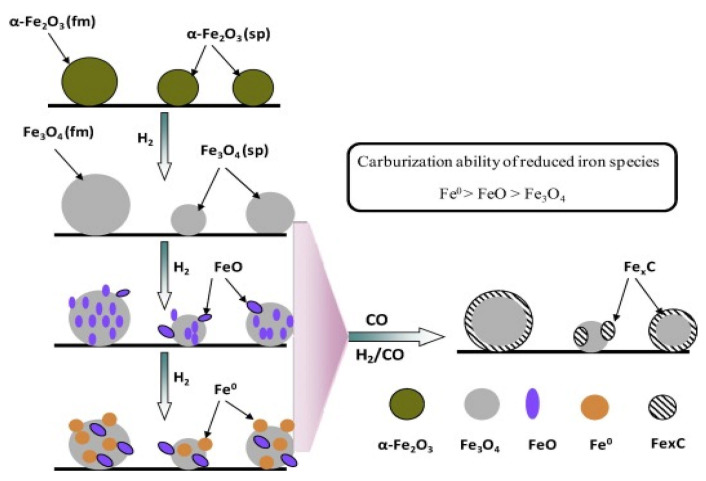
Carburization behaviors of different reduced iron phases for the iron-based catalyst. Reproduced from Ref. [42] with copyright license from Elsevier (2021).

Iron-based catalysts tend to form various phases during the course of the reaction, such as χ-Fe (Fe^0^), χ-Fe_2_O_3_, (hematite), Fe_3_O_4_ (magnetite) and different forms of iron carbides with carbon atoms in octahedral interstices (O-carbides: χ-Fe_2_C, χ’-Fe_2.2_C and Fe_x_C) and trigonal prismatic interstices (TP-carbides: χ-Fe_2.5_C and χ-Fe_3_C) [22,23,24]. In general, the fresh Fe catalyst is mainly formed by α-Fe_2_O_3_, which can be converted into different reduced iron species upon reduction with hydrogen (Figure 4). Considering the study by Ding et al. [42], the reduction with H_2_ of the iron phases located in the bulk and on the catalyst surface follows the sequences: α-Fe_2_O_3_ → Fe_3_O_4_ → FeO → α-Fe(0), while their carburizing ability follows the order: α-Fe(0) > FeO > Fe_3_O_4_. This means that iron carbides are formed from Fe(II) species. By considering this trend, an equilibrium between iron carbides and Fe(II) oxide species during the CO_2_ hydrogenation process is expected. Assuming that there is a unique presence of iron carbides (especially for χ-Fe_5_C_2_) on the catalyst surface, there is a gradual formation of hydrocarbon species on the surface of the iron carbides. 

It is known that iron oxide is more effective for the RWGS reaction, while Hägg carbide (χ-Fe_2.5_C) species and polymeric surface carbon species (C_β_) are active in the FTS reaction [43]. In fact, there is experimental evidence that the RWGS reaction and CO_2_ activation could occur on the surface of iron oxides and iron carbides, respectively [44]. For FeOC_x_ nanocrystals, oxygen vacancies and carbon vacancies operating in the RWGS and FTS reactions, respectively, play an important role [45]. Therefore, modification of the reaction rate and reaction pathway can be achieved by optimizing the amount of oxide and carbide phases by adjusting the reducibility of the iron species. The most active Fe phase in FTS has not been unequivocally identified yet because of the absence of effective in situ characterization techniques. In this sense, the similar product selectivity under steady-state conditions for CO and CO_2_ hydrogenation reactions strongly suggests that both reactions could occur at the same catalyst active sites [19,46]. However, the adsorption rate of CO_2_ is slower than that of CO, which explains the lower conversion of CO_2_, the higher degree of hydrogenation of the intermediates and the easier formation of CH_4_ [46].

**Figure 5:**
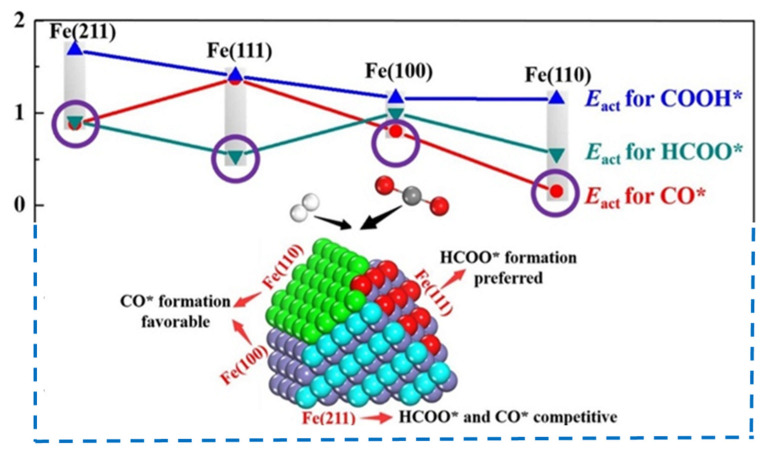
Energetically favorable adsorption configuration of CO_2_ and H_2_ on the Fe(100), (110), (111) and (211). Reproduced from Ref. [36] with copyright licence from Elsevier.

In the CO_2_ hydrogenation reaction, both direct CO_2_ activation and H-assisted CO_2_ activation are of great importance. The CO_2_ and H_2_ adsorption modes were studied by Wang et al. [36] using periodic spin-polarized density function theory (DFT). By analyzing the most energetically stable CO_2_ and H_2_ adsorption configurations, it was concluded that the Fe(110) facet is the most promising candidate for CO_2_ hydrogenation due to its lower barrier for HCOO* formation and its higher ability to activate CO_2_; CO_2_ dissociation to CO* could occur on the Fe(111) and Fe(100) facets, while the kinetically competitive formation of CO* and HCOO* occurs on the Fe(211) facet (Figure 5) [36]. Similarly, the CH*x coupling mechanism proposed by Pham et al. assumes that chemosorbed CO_2_ can dissociate directly to CO* at the Fe(100) facet, and then the chemosorbed CO* dissociates to C, which hydrogenates to monomer CH* [47].

The facet effect on CO_2_ adsorption, dissociation and hydrogenation on the thermodynamically stable χ-Fe_5_C_2_ (510) and θ-Fe_3_C (031) facets were also investigated by Liu et al. [48]. Their DFT calculation demonstrated that CO_2_ direct dissociation occurs on both χ-Fe_5_C_2_ and θ-Fe_3_C phases (*E_a_* = 0.17 eV). The one-step formation of *CO + *OH was proven to be feasible on χ-Fe_5_C_2_ (*E_a_* = 0.24 eV), whereas the *HCOO pathway (*E_a_* = 0.20 eV) and *CO + *OH formation (*E_a_* = 0.11 eV) was favored on the θ-Fe_3_C phase. Interestingly, the DFT calculation suggests that neither χ-Fe_5_C_2_ (510) nor θ-Fe_3_C (031) facets favor the formation of *COOH [48].

**Figure 6:**
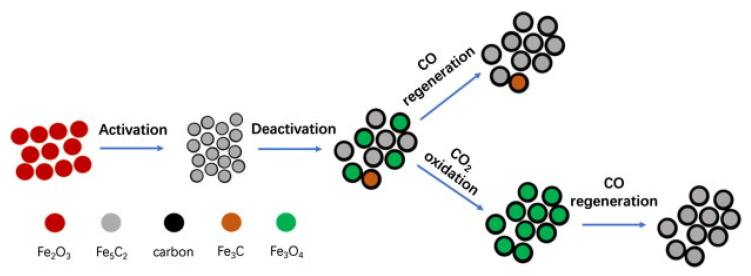
Transformation of iron phases during the life cycle of the catalyst tested in the selective olefin formation reaction from CO_2_. Reproduced from Ref. [41] with copyright license from Elsevier (2021).

The iron phases transition during the activation → reaction → deactivation → regeneration life cycle of the iron catalyst was investigated by Zhang et al. [41]. After the whole life cycle (Figure 6), the catalyst still showed 91.8% of the initial activity and 97.7% of its initial selectivity toward C_2_–C_4_= olefins. By using the operando techniques, the following phases transitions occur: the catalyst activation (Fe_2_O_3_ → Fe_5_C_2_ (catalyst activation), Fe_5_C_2_ → Fe_3_O_4_ (reaction and deactivation) and (Fe_3_O_4_ → Fe_5_C_2_) regeneration. The correlation between the catalyst activity and the types of phases formed suggested that the Fe_5_C_2_ phase was responsible for the formation of C_2_^=^–C_4_^=^ olefins. Since the iron carbide phases were irreversibly oxidized to Fe_3_O_4_, the loss of this phase was the main factor leading to catalyst deactivation (Figure 6) [41].

## 4. Promotion of Fe-Based Catalysts

A general limitation of the modified FT synthesis process is the low selectivity to light olefins, and this problem cannot be solved by using only monometallic Fe catalysts. This is because, at a low reaction temperature, there are some limitations of the FTO process, such as high selectivity to methane and rapid deactivation. In addition, during the in situ activation of the catalyst, the formation of different types of iron species occurs [22,23,24]. Obviously, the main objective of direct hydrogenation of CO_2_ to light olefins is to maximize selectivity towards light olefins and minimize methane production.

In order to modify the number of CO_2_ adsorption sites, the effective method is the addition of promoters to iron- or cobalt-based catalysts. The most frequently employed promoters are metal oxides (ZnO, MnO), metals (Cu, Ru), alkali metals (K, Na, Cs, Rb) and alkaline earth metals (Ca, Mg). The latter promoters play a positive role in CO_2_ adsorption and in modulating the electronic property of the catalyst. Stabilization of small metal nanoparticles could be obtained by the addition of a structural promoter such as silica to improve the specific area of the catalyst and its attrition resistance. In addition, the structural promoter might influence the activity and selectivity in CO_2_ hydrogenation by changing the catalyst acidity and enhancing active phase dispersion on the support surface. In fact, the addition of silica binder to the iron catalyst prepared by precipitation is usually performed when the CO_2_ hydrogenation is carried out in a fixed bed reactor. Similarly, mesoporous materials are promising candidates due to their high surface areas and large pore sizes.

### 4.1. Promotion with Co

A commercial Fischer–Tropsch catalyst is known to contain mainly iron and cobalt. Their simultaneous presence on the catalyst surface is complementary to each other, as both metals have distinct merits and defects. In contrast to cobalt, which remains in the metallic state during the FTs process, iron-based catalysts tend to form several phases during the course of the reaction. The effect of Co was intensively studied [49,50,51,52,53,54,55,56,57,58]. The experimental results suggest that the use of Co as a promoter can accelerate the CO consumption from the RWGS reaction, leading to an increase in CO_2_ conversion over Fe-based catalysts [46,50,51,52,53,54,55,56,57,58]. For example, the benefit of iron and cobalt co-presence in an FTS catalyst was studied by Yang et al. [49]. The Co/Fe_5_C_2_ catalyst was prepared by decorating the Fe_5_C_2_ base catalyst with a small amount of Co metal particles via a secondary crystal growth process (Fe/Co = 12). Optimization of the Co content by adjusting the Fe/Co molar ratio from 3.3 to 25 showed that the optimum Co loading was very small (0.6 wt.% Co). The bimetallic Fe_5_C_2_/Co shows excellent catalytic activity in FTS at low temperatures (CO + H_2_) while maintaining the characteristics of the iron carbide components in terms of chain propagation and termination behavior. The experimental data combined with DFT calculation reported by Yang et al. suggested that CO (CO_2_) activation might occur at Co sites while hydrogenation and chain growth take place at Fe_5_C_2_ active sites [49]. A significant enhancement of the C_2+_ hydrocarbons formation over Fe–Co/Al_2_O_3_ bimetallic catalysts was achieved by utilizing a low Co content (Co/(Co + Fe) atomic ratio of 0.17) [50]. This catalyst was even more effective for synthetizing light olefins after its modification with a high amount of K [51]. Similarly, doping the Fe_2_O_4_ catalyst with Co enhances the selectivity toward C_2+_ hydrocarbons in CO_2_ hydrogenation due to the intimate contact between Co and Fe, leading to higher selectivity toward light olefins [52]. In addition, the enhanced dispersion of both phases accelerated the formation of long-chain products and helped inhibit methane formation [52]. The high dispersion and intimate contact between Fe and Co sites inhibited methane formation and favored higher selectivity of the light olefins. With respect to methane, the study by Jimenez et al. showed that its formation could be avoided by adapting the surface orientations of the Co_3_O_4_ catalysts [53]. Interestingly, the recent study by Calderone et al. demonstrated that the core-shell structure with an iron core and a cobalt shell are effective catalytic systems for the production of olefins due to a cooperative effect between both metals [54].

Recently, CO_2_ was effectively converted to light olefins through a hydrogenation reaction over Na-promoted Fe–Co/NC catalysts derived from ZIF-67 [55]. The Fe–Co catalyst supported on N-doped carbon (NC) was prepared by pyrolysis of pre-synthesized Fe–Co/ZIF-67 at temperatures above 500 °C. This preparation method effectively anchored the metal particles resulting in uniform dispersion of the active sites. The presence of Na also promotes chain growth and suppresses the direct hydrogenation of Fe-(CH_2_)n intermediates. Furthermore, the N_2_ pretreatment of the Na-promoted Fe–Co/NC catalysts led to the formation of Fe–Co alloy, Fe_3_O_4_, Fe_5_C_2_ and Co_2_C species [55]. The catalyst N_2_ pyrolysis at 600 °C led to the best FeCo/NC-600 catalyst exhibiting the highest selectivity toward light olefins (27%) at a CO_2_ conversion of 37%. From the catalyst activity–structure correlation, it was concluded that it was probably due to the combination of suitable particle size and sufficient active sites of iron carbide. This novel bifunctional hybrid catalyst prepared by Dong et al. shows good stability during 50 h of the reaction run [55]. As a summary, Table 1 compares the general futures of the performance and properties of Fe and Co–Fe catalysts in the CO_2_ hydrogenation reaction.

### 4.2. Promotion with Alkali Metals

**Figure 7:**
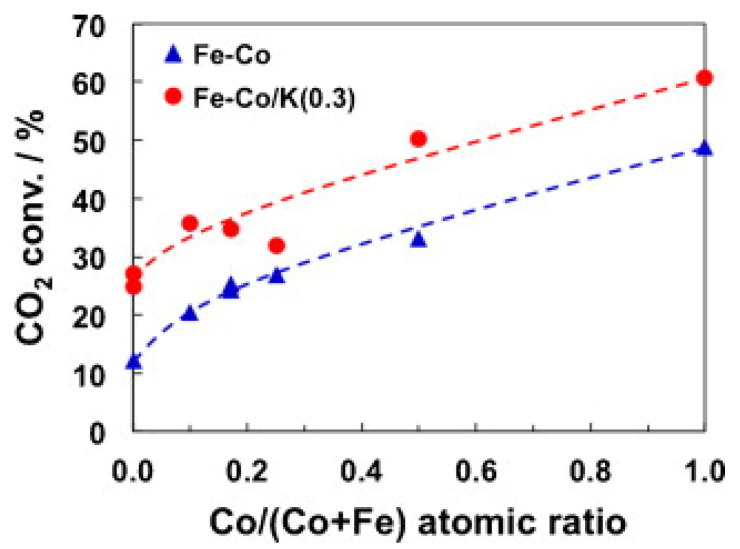
Effect K promotion of Fe–Co/K/Al_2_O_3_ catalysts on CO_2_ hydrogenation. Reproduced from Ref. [50]. Copyright license from Elsevier (2021).

The enhancement of both CO_2_ conversion and the selectivity toward C_2_–C_7_ hydrocarbons can also be achieved by the addition of a small amount of the alkali metals [59,60,61,62,63,64,65,66,67,68,69,70,71,72,73,74,75,76,77]. This is due to the increased basicity of the catalyst, inhibition of H_2_ dissociative adsorption and enhancement of iron carbide formation. In addition, there is an inhibition of methane formation and an increase in the chain growth probability. Potassium is probably the most studied promoter for iron catalysts because it enhances CO_2_ chemisorption and inhibits H_2_ chemisorption, affecting thus the relative surface coverage of reactants. This is due to suppression of the hydrogen by electron-donating potassium, as hydrogen itself donates an electron to Fe upon adsorption [59]. Therefore, changes in the chemisorption properties of the metal surface toward the reactive molecules are induced by geometric and electronic-type effects of the promoters [60]. As an example, Figure 7 shows the enhancement of the CO_2_ conversion of Fe–Co/K/Al_2_O_3_ catalysts with respect to catalyst without K [50], which can be explained considering the enhancement of the basicity of the catalyst surface leading to easy desorption of olefin products [73].

The presence of alkali on the catalyst surface has geometrical and electronic effects deduced from the changes in the chemisorption capacity of the reactants on the metal surface [60]. This is because there is a charge transfer from the alkali metal (electron donor species) to the surface of the catalyst, thereby enhancing the chemisorption of electron acceptor species, such as CO_2_ and oxygen, and inhibiting the chemisorption of electron donor species, such as H_2_ and olefins. However, for both CO_2_ conversion and product distribution, doping levels must be optimized because over-doping leads to the suppression of desired hydrocarbon products, as was demonstrated by Doner et al. [61] for CO_2_ hydrogenation over Mn- and K-doped Fe/Al_2_O_3_ catalysts. Their Mn–Fe/Al_2_O_3_ catalysts doped with an optimized amount of K exhibited a CO_2_ conversion of about 40% and an olefin/paraffin ratio greater than four. From the activity–structure correlation, it was concluded that KAlH_4_ could be part of the active phase acting as an H_2_ activation center and reversible H_2_ reservoir [61]. In fact, the inhibition of olefin hydrogenation is generally considered to be the main reason for the increase in the olefin-to-paraffin ratio [37]. However, DFT calculations suggest that the olefin-paraffin ratio may be more influenced by olefin desorption than by olefin hydrogenation [37].

As in the case of K, it was observed that increasing Na promoter led to an increase in CO_2_ conversion and selectivity towards olefin formation [71]. However, there is a certain Na loading limit where both parameters reached a plateau (the highest CO_2_ conversion and olefin selectivities were 36.8% and 64.3%, respectively) [62,63]. In this regard, Liang et al. observed that Na content influenced the amount of the active phase, enhanced the adsorption of CO_2_ on Fe_5_C_2_ and its stability and inhibited the secondary reaction of alkene hydrogenation [62,63,71]. The increase in both CO_2_ conversion and selectivity to light olefins are generally explained as due to combined effects of the catalyst basicity and the enhancement of CO adsorption and active phase carbonization [62,63]. The addition of the Na promoter led to a decrease in particle size of the Fe_5_C_2_ active phase, which in turn favors the inhibition of hydrogenation of intermediate carbon species to paraffin. Compared to the Na-free catalyst, the Na catalyst (0.5 wt.% Na) has a higher olefin-to-paraffin ratio (5.67 vs. 0.70) and an exceptionally high yield of light olefins (24.7%) (Table 2). In addition, the increase in the density of Na nanoparticles on the Fe_5_C_2_ surface improved the catalyst stability by suppressing the hydrogenation of those species [63]. A comparison of the literature data is difficult because the reaction conditions and reactor types used are different. In addition, catalyst activity and selectivity are highly dependent on catalyst formulation, metal loading, preparation procedure, type of solvent, etc. The promoting effect of the selected catalytic systems is clearly seen in Table 2, which shows how the light olefin yields depend on the reaction conditions and catalyst formulation.

Even a very small amount of Na could enhance the production of C_2_^=^–C_4_^=^ olefins and C_5+_ hydrocarbons, as demonstrated by Wei et al. [65,66] for Fe_3_O_4_-based nanocatalysts containing residual Na. Their Na-containing iron catalysts were prepared by a simple one-pot synthesis method using NaOH not only as a precipitating agent but also as a promoting source. Besides a very low Na content, Na was an effective promoter for decreasing methane selectivity, favoring chain growth propagation and increasing the olefin/paraffin ratio of the products. This was because the presence of Na improved the surface basicity, which in turn enhanced olefin production and carbonization of its iron phases. As a consequence, Na-containing iron catalysts showed higher activity and produced more C_2=_–C_4=_ olefins and C_5+_ hydrocarbons than their Na-free counterparts. The results strongly suggested the synergistic effect between sodium and iron inhibiting the secondary hydrogenation of olefins and accelerating the chain growth reaction, producing more C_2_^=^–C_4_^=^ and C_5+_ hydrocarbons. The best activity and selectivity results were obtained with the catalyst prepared with a Na/Fe weight ratio of 1.18/100. At a CO_2_ conversion of 40.5%, this catalyst exhibited a high olefin/paraffin ratio (6.2) and high selectivity to C_2=_–C_4=_ (46.6%). It was hypothesized that the presence of Na increased the dissociative adsorption rate of CO, leading to the inhibition of olefin readsorption at the active sites due to the increased surface coverage of dissociated CO. As a consequence, the selectivity towards olefins and heavy hydrocarbon products increased. The recent study by the same authors on the evolutions of Na-modified carbon and iron species and their tuning effect on the hydrogenation of CO_2_ to olefins confirmed this hypothesis [65,66]. Table 2 compares the olefin yields in CO_2_ hydrogenation over the selected catalysts. As seen in this table, the combined effects of double promotion with K and Co together with the effect of the pore size of the support was an effective strategy for olefin production [51].

### 4.3. Promotion with Mn

Among the different promoters, the use of manganese oxide as a promoter was one of the most investigated because its presence on the support surface positively affected the physicochemical properties of the iron catalyst [68,78,79,80,81,82,83,84,85]. In particular, the enhancement of reducibility and carburization of Fe_3_O_4_ NPs, and the modification of the surface exposure of iron species were observed [19]. As a consequence, the Mn-promoted catalysts were more active than their Mn-free counterparts and showed a lower hydrogenation capacity manifested by the suppression of C_5+_ hydrocarbon formation. This is probably due to the easier desorption of olefins from the active sites, as suggested by the DFT calculation [37].

For example, improved selectivity towards light olefins was observed after doping of the Fe catalysts with Mn up to 15 wt.% [82,83]. In those systems, Mn acted as a structural and electronic promoter interacting with Fe_3_O_4_ nanoparticles [82,83].

**Figure 8:**
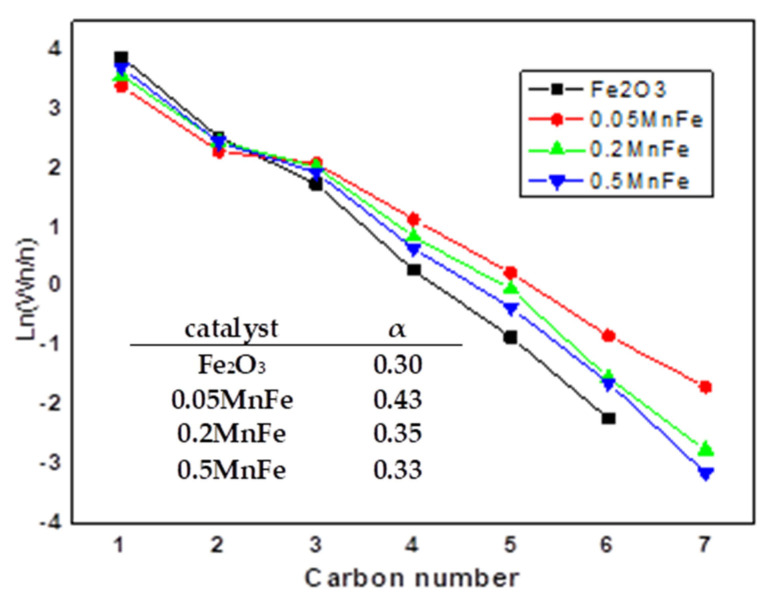
Anderson–Schulz–Flory distribution of the hydrocarbons in the CO_2_ hydrogenation over bulk *x*MnFe catalysts. Reproduced from Ref. [19]. Copyright license from Elsevier (2021).

The effect of Mn promotion of Fe catalyst on the olefin formation from CO_2_ was investigated by Al-Dossary et al. [19]. The bimetallic MnFe catalysts were prepared with varied Mn-to-Fe molar ratios by the sol–gel method using a triblock copolymer as a structure-directing agent. It was found that a lower concentration of H_2_ than CO_2_ could be favorable for olefin formation via CO_2_ hydrogenation at 340 °C and 20 bar. As a consequence of its best textural property, the mesoporous 0.05MnFe (Mn/Fe molar ratio of 0.05) exhibited the best catalytic performance with improved selectivity to C_2_–C_5_ and C_6+_ hydrocarbons, the formation of some oxygenates and the reduced CO and CH_4_ formation [19]. The effect of Mo loading on the product distribution of hydrocarbons was calculated using the Anderson–Schulz–Flory (ASF) Equation (1). The experimental results are shown in Figure 8, while the chain growth probability (α) calculated from the linear part of the ASF plots is listed in the inlet table of this figure. As seen, the most active 0.05MnFe catalyst exhibits the greatest chain growth probability among the catalysts studied.

**Figure 9:**
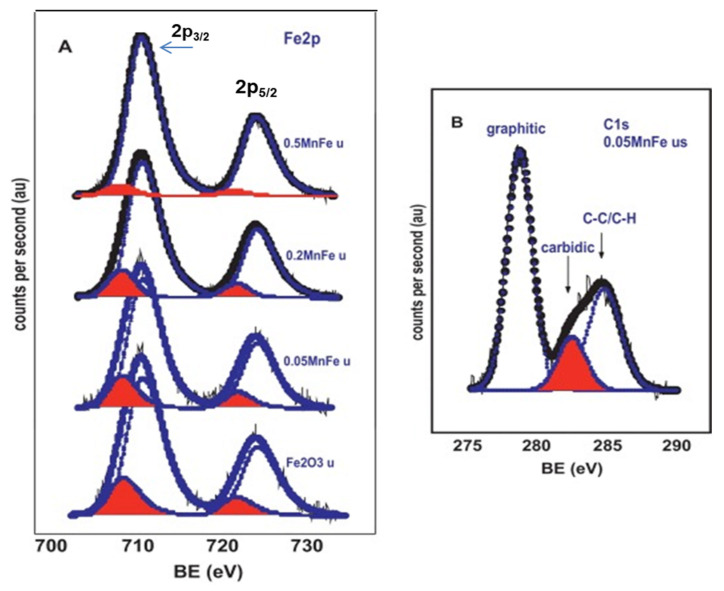
(**A**) Fe 2p core-level spectra of spent MnFe catalysts tested in the CO_2_ hydrogenation. The red color refers to iron carbide species. (**B**) C 1s spectrum of the most active 0.05MnFe catalyst showing different carbon species. Reproduced from Ref. [19] with copyright license from Elsevier (2021).

Regardless of the Mn content, Hägg carbide (χ-Fe_5_C_2_) was the main iron carbide phase developed on the surface of MnFe catalysts, as confirmed XPS characterization of the spent catalysts (Figure 9) [19]. However, the formation of other iron carbides was not excluded because of very similar binding energies of the most intense Fe 2p_3/2_ line of the Fe 2p doublet. The catalyst doping with high Mn content (Mn/Fe ratios >0.5) exhibited increasing CH_4_ formation and decreasing CO_2_ conversion due to segregation of manganese oxide, limiting the accessibility of reactants to the Hägg carbide phase. It was concluded that the coverage/blocking of the Hägg carbide phase by amorphous MnO_x_ species could be minimized by designing the catalyst with a mesoporous structure [19].

Recently, Jiang et al. [78] prepared an efficient catalyst for olefin production from CO_2_ by decorating magnetite microspheres with nanoparticles of manganese. Microspheres of magnetite (Fe_3_O_4_) were prepared by hydrothermal method, and their doping with Mn NPs was performed by incipient wet impregnation method. By optimizing the promoter dispersion and its interaction with magnetite microspheres, the most optimized 10Mn–Fe_3_O_4_ catalyst showed high CO_2_ conversion (44.7%) and selectivity towards C_2_–C_5_ olefins (46.2%), yielding 18.7% of light olefins (Table 2). The catalyst characterization suggested that the role of Mn as a promoter was to facilitate CO_2_ adsorption, promote C–O bond activation and inhibition of secondary hydrogenation [78].

### 4.4. Others Promotors

The effect of the iron catalysts doping with Cu, V, Zn, Mg, N and Ce was also investigated [11,45,86,87,88,89,90,91,92]. For example, enhanced light olefin production was observed after nitrogen doping of the Fe catalysts supported on carbon nanotubes [11]. The Fe/NCNTs catalyst synthetized by Lu et al. showed high activity and stability in FTO reaction with high selectivity for light olefins (up to 46.7%) [11]. It was hypothesized that the formation of χ-Fe_5_C_2_ was induced by nitrogen, while the enhanced catalytic activity was explained as due to improved dissociative adsorption of CO and inhibition of olefin hydrogenation.

The effect of the Fe/K–Al_2_O_3_ catalyst doping with different transition metals was studied by Chaipraditgul et al. [86] and Landau et al. [45]. The positive effect was observed for catalyst promotion with Mo and Zr [86], while the promotion with Ce, Zr and Cu increases selectivity to higher C_5_ hydrocarbons [82]. In the case of Cu-promoted catalysts, the high hydrogenation of secondary olefins was originated by the high ability of Cu to dissociate H_2_ and its high intrinsic hydrogenation capacity. A strong promotion effect of Cu on the formation of olefin-rich C_2_+ hydrocarbons was observed in the CO_2_ hydrogenation over Fe–Cu/γ-Al_2_O_3_ catalysts [87]. In addition, the suppression of methane formation was achieved with the catalyst with optimized Cu content (Cu/(Cu+Fe) atomic ratio of 0.17). With respect to Zn, its effect is still unclear, as a positive product shift towards lower hydrocarbons was reported by Zhang et al. [35], while the favored formation of longer C_5+_ hydrocarbons was observed by Chaipraditgul et al. [86].

As with Zn, the effect of Ce is unclear. This is probably because ceria promotes both WGS and RWGS reactions, which is a critical requirement for activating CO_2_ molecules [24,25]. The enhancement of the formation of olefins after modification of SAPO-34 zeolite with ceria was observed by Ghasemi et al. [88]. In contrast, the study by Fierro and co-workers showed that catalyst doping with ceria had little effect on the activity and selectivity of unsupported Fe and Fe–Mn catalysts [89].

Under the same reaction conditions, both Ce-free and Ce-containing iron catalysts showed similar conversion levels yielding hydrocarbons (C_1_–C_10_) with high selectivity [89]. The positive effect of ceria was the shortening of the induction period for hydrocarbon formation via CO_2_ hydrogenation due to a higher carburization rate of the Fe catalyst promoted by the Ce. This higher reaction rate was explained as due to the formation of easier to dissociate tilted CO species. Since Ce (III) species are required in the catalytic framework to form such tilted CO species, their presence was confirmed by X-ray photoelectron spectroscopy [23].

**Figure 10:**
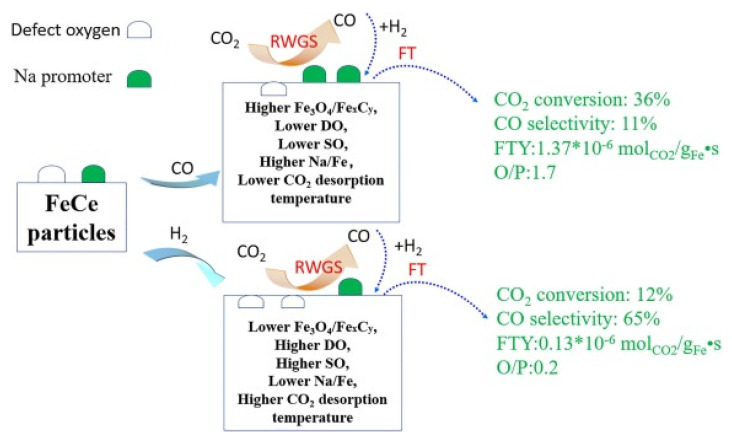
The effect of reducing gas atmosphere (CO_2_ vs. H_2_) on the CO_2_ hydrogenation over 3% FeCeNa catalyst. Reproduced from Ref. [90] with copyright license from Elsevier (2021).

The effect of the reducing atmosphere (CO versus H_2_) on the efficiency of 3%CeFeNa catalyst toward olefin formation was investigated by Zhang et al. [90]. It was found that the catalytic performance strongly depends on the presence of CO and H_2_ in the gas atmosphere (Figure 10). Changing the gas atmosphere from CO to H_2_, the CO_2_ conversion decreased from 36% to 12%, and the olefin-to-paraffin ratio decreased from 1.7 to 0.2. The characterization of the spent catalyst by different techniques showed that this could be linked to the observed decrease in the Fe_3_O_4_/Fe_x_C_y_ and Na/Fe ratios of the catalyst tested in the H_2_ gas atmosphere. It was hypothesized that this is because the CO_2_ adsorbed on the catalyst surface reacts with H_2_ producing a higher amount of the surface oxygen ions. Their presence on the catalyst surface inhibits the conversion of CO_2_ to CO and the transformation of CO to hydrocarbons [90].

In summary, a typical Fe-based catalyst for FTO consists of a bulk Fe oxide promoted by several elements. Potassium is the best electronic and structural promoter catalyzing the RWGS reaction, improving CO_2_ conversion and selectivity towards large hydrocarbons and olefins and decreasing methane yield. Its presence in the catalyst increases the basicity of the catalyst surface, improves the dissociative adsorption of CO and inhibits the dissociative adsorption of H_2_. Copper and manganese are also structural and electronic promoters, which increases the reduction and carburization rates (thus forming a larger number of active sites). The Cu-doped catalyst shows higher CO_2_ conversion and higher yields of light olefins. The addition of zinc as a structural promoter also modified the structure and surface adsorption behavior of Fe catalysts, leading to the improvement of CO_2_ adsorption [21], promotion of hydrogen dissociation, acceleration of the FTS reaction and improvement of selectivity towards olefins and stability of the catalysts. Zinc is known to be very active in WGS and RWGS reactions [19].

## 5. Tandem Catalytic Systems

For the effective olefin formation via FTO or MTO routes, the promising strategy should be the design of tandem catalysts [3]. This is because these catalysts could couple multiple reactions on a single catalyst. In addition, its use for CO_2_ hydrogenation allows CO_2_ activation under mild reaction conditions along with control of C–C coupling for enhancement of olefin formation and suppression of undesirable CO and CH_4_ formation.

**Figure 11:**
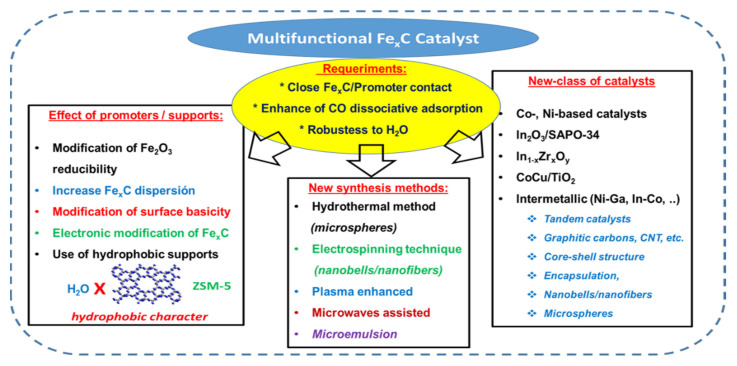
The catalyst requirements for effective C_2_–C_4_= olefin production from CO_2_ and the strategies for the preparation of the optimized Fe_x_C-based catalyst.

Recently, the development of tandem catalysts for the hydrogenation of CO_2_ to olefins was reviewed by Ma and Porosoff [3]. In addition to the metallic function, the essential part of the tandem catalyst is the zeolite [93]. This is due to the unique morphology of zeolites, such as ZSM-5 H-Beta, SAPO-34 and to their strong acidity that allows the cracking of larger hydrocarbons into olefins. This is because both porous aluminosilicates exhibit high specific surface area, regular crystalline framework structure, moderate acidity and hydrophobic character [26]. SAPO-34 molecular sieve exhibit a hierarchical structure of chabazite topology and narrow pore opening (0.38 × 0.38 nm), while ZSM-5 zeolite has a two-dimensional pore system with straight (0.53 × 0.56 nm) and sinusoidal (0.51 nm × 0.55 nm) interconnected channels. It is known that zeolite ZSM-5 favors either C_5_+ or aromatics hydrocarbons formation, while SAPO-34 demonstrated to favor light olefin production via-methanol mediated reaction pathway. In comparison to γ-Al_2_O_3_, both zeolites exhibit better tolerance to adsorption of water, and in consequence, better catalytic activity [26]. The strategies for the preparation of the optimized Fe-based catalysts are presented in Figure 11.

For the FTO reaction route, a good example of a tandem catalyst is the CeO_2_–Pt@mSiO_2_–Co core catalyst prepared by Xie et al. [94]. In this catalyst, RWGS and FTO reactions occur at the interfaces between the core (CeO_2_–Pt) and the shell (Co-doped mesoporous silica (mSiO_2_–Co). It should be noted that the CO formed in the core by the RWGS reaction was fully hydrogenated by the FTO reaction to hydrocarbons in the mSiO_2_–Co layer because this layer was not active for the RWGS reaction.

The decrease of CO_2_ formed during the direct synthesis of light olefins via the FTO reaction is a major challenge for Fe-based FTO catalysts. Recently, this was achieved with the FeMn@HZSM-5 capsule catalyst with FeMn in the core and HZSM-5 as the shell [95]. Compared to the FeMn catalyst, the capsule catalyst showed much better catalytic behavior with enhanced selectivity towards light olefins, and CO_2_ selectivity decreased by more than 10%. In addition, the capsule structure of HZSM-5 was found to suppress the WGS reaction due to the effective diffusion of H_2_O. This enhancement of the catalyst behavior towards the production of light olefins by the FTO route is due to the fact that capsule catalysts are bifunctional, and their catalytic behavior was found to always be better than that of the corresponding catalysts prepared by physical mixing [95].

**Figure 12:**
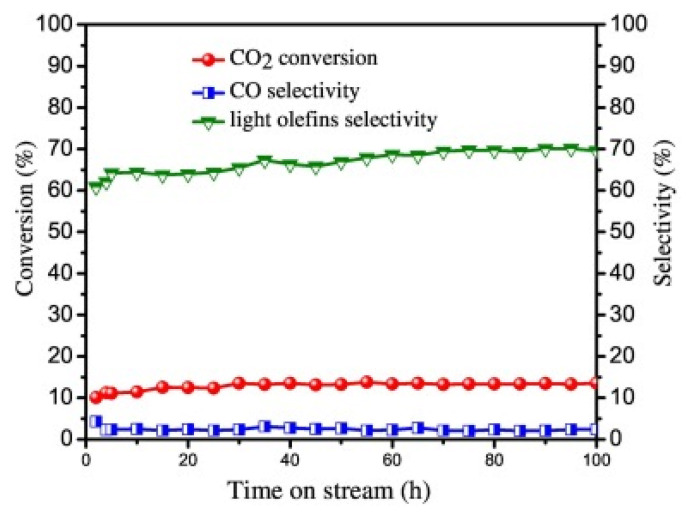
Catalytic behavior of the In_2_O_3_/ZrO_2_&SAPO hybrid catalyst in CO_2_ hydrogenation during long-term activity test. Reaction conditions were: 300 °C, 2 MPa and GHSV of 2160 cm^3^ h^−1^ g_cat_^−1^. Reproduced from Ref. [97] with copyright license from Elsevier (2021).

The break through the Anderson–Schulz–Flory distribution of the CO-mediated reaction route could be achieved using an oxide–zeolite composite catalyst. Interestingly, compared to the hybrid metal oxide/SAPO-34 catalyst, the Fe catalysts offer lower selectivity toward light olefins. In this regard, there are works demonstrating that it is possible to directly convert CO_2_ into light olefins using bifunctional catalysts, such as a hybrid catalyst composed with In_2_O_3_/ZrO_2_ and SAPO-34 [8,96,97]. The high selectivity of the In_2_O_3_/ZrO_2_&SAPO hybrid catalyst towards olefin formation (~77.6%) (Figure 12) demonstrated that the breaking of the Anderson–Schulz–Flory distribution is possible by proper catalyst design. Unfortunately, the yield of olefins is still low (about 8%) [98].

Despite intensive work on tandem bifunctional catalysts, poisoning of the catalyst by the produced CO (by-product) remains an unsolved problem [96]. In this regard, suppression of CO adsorption on active sites was recently reported for the tandem In_2_O_3_/ZrO_2_&SAPO catalyst combining metal oxide (In_2_O_3_/ZrO_2_) and acid (SAPO-34) functions [96,97]. The important feature of this catalyst is the absence of deactivation during 100 h of current operation by adsorption of CO and H_2_O on the active sites (Figure 12). The olefins were formed on the same catalyst via the MTO reaction pathway: first, methanol was formed on the oxygen vacancies of the metal oxides (In_2_O_3_/ZrO_2_) and then transformed inside the zeolite channel into light olefins [97].

As compared with the CO-mediated route, the olefin formation via the methanol-mediated route is more difficult. In fact, the high selectivity toward light olefins (up to 80%) can be achieved only at low CO_2_ conversion (about 13%). Higher CO_2_ conversions lead to lowering of olefins selectivity due to side reactions [39]. The catalysts are bifunctional with the metal function necessary for the hydrogenation of CO_2_ to methanol and the acid function, provided by HZSM-5 or SAPO-34 zeolites, which is required for the transformation of methanol to light olefins [39,96,97,98,99,100,101,102].

**Figure 13:**
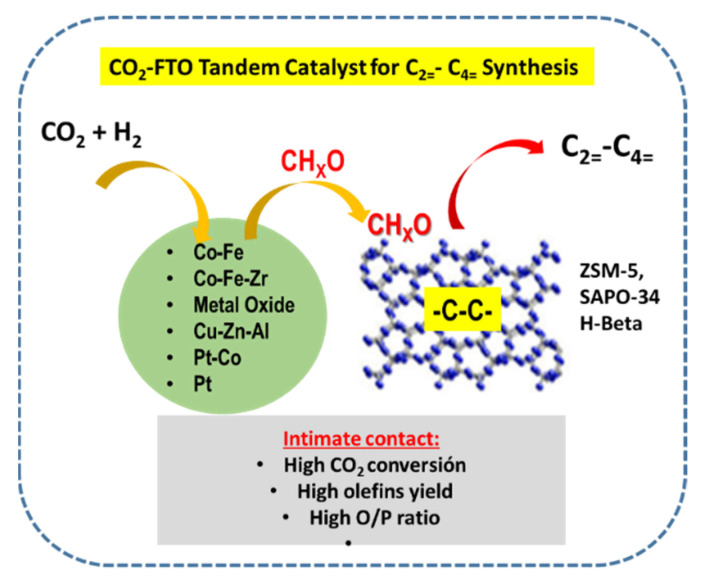
Visualization of the reaction mechanism of the direct hydrogenation of CO_2_ to olefins over tandem catalyst.

Among different zeolites, the best selectivity results were obtained with SAPO-34 zeolite. This is because of its favorable framework structure with a CHA cage (9.4 Å) and mild acidity, allowing the optimum intracrystalline diffusion path length and the optimum mild acidity [103]. By controlling the catalyst synthesis conditions, the yield of light olefins and the catalyst lifetime can be improved [104,105,106,107,108]. In this regard, the catalyst with a core-shell structure with an iron core and a cobalt shell proved to be effective for olefin formation due to a cooperative effect between the two metals and shape selectivity. The excellent results were also reported by Ghasemi et al., who designed a highly efficient hybrid catalyst composed of NiCu/CeO_2_ and SAPO-34 [88]. The synthetized NiCu/CeO_2_-SAPO-34 demonstrated to covert CO_2_ directly into light olefins with selectivity up to 76.6% at CO_2_ conversion of 15.3%. With no apparent loss of activity, this hybrid catalyst was stable over a reaction time of 90 h. In this catalyst, methanol was produced on the NiCu/CeO_2_ surface, while methanol conversion to olefins occurred within the porous structure of the SAPO-34 zeolite [88]. The typical method of the preparation of the hybrid catalyst is visualized in Figure 13.

The high water production in the RWGS reaction could reduce the selectivity towards low olefins and could damage the structure of the acid catalysts. This is because the adsorption of H_2_O on the catalyst surface negatively affects the functionality of metal and acid sites. In particular, the strong adsorption of water on the hydrogenation sites inhibits the production of olefins via the MTO route of the CO_2_ reaction due to the blocking of the methanol production sites. Therefore, direct olefin formation requires optimization of reactor designs to remove water in situ, e.g., using membranes or distillation [105] and the design of water-resistant and highly selective catalysts.

The enhancement of selectivity toward light olefins was observed after the addition of various amounts of zirconium into bifunctional In_2_O_3_/SAPO-34 catalyst [97,99,100,102]. For the In_1-x_Zr_x_O_y_/SAPO-34 systems, the addition of an optimized amount of Zr markedly enhanced catalytic behavior leading to selectivity for C_2_–C_4_= as high as 65–80% with only about 2.5% methane at CO_2_ conversion of 15–27% at the relatively high reaction temperature of 380 °C. The product distribution was very different from that expected from the Fischer–Tropsch synthesis route and Anderson–Schultz–Flory distribution. The combined experimental and computational DFT calculation performed by Dang et al. [97] suggested that reaction intermediates could be adsorbed at the defect surface sites of In_1-x_Zr_x_O_y_ solid solution, whereas CO_2_ chemisorption occurs at the oxygen vacancy created on the surface of zirconium oxide having sites with higher binding energy than that of In_2_O_3_ surface. Noticeably, the CO formation was suppressed; therefore, the RWGS reaction did not occur [97].

It is well known that the catalyst textural and acid properties have great importance for the final catalyst behavior. In a recent study on the atmospheric pressure CO_2_ hydrogenation to olefins, Gupta et al. [103] observed that the ordered mesoporous structure of Co_3_O_4_ catalysts favored the chain growth of carbon atoms for the production of C_2+_ hydrocarbons while non-porous Co_3_O_4_ nanoparticles showed strong selectivity toward CH_4_. The ordered mesoporous cobalt oxides were synthesized using 3D KIT-6 and 2D SBA-15 as silica templates to obtain the catalysts with three-dimensional and two-dimensional pore morphology, respectively. High selectivity for C_2+_ (∼25%) was obtained for both 3D and 2D types of morphologies, but the Co_3_O_4_ catalyst with 3D pore structure formed more olefins (54.9%). Superior CO_2_ hydrogenation activity and selectivity achieved with the 3D mesoporous Co_3_O_4_ was attributed to enhance of the number of active sites and the lowering of mass diffusion limitations due to 3D catalyst pore structure [105]. The former component of this composite is a methanol synthesis catalyst, whereas the latter is a methanol-to-olefins catalyst. The influence of the physicochemical properties of Fe–Co/K–Al_2_O_3_ catalysts on their activity and selectivity towards olefin formation was investigated by Numpilai et al. [107]. In good agreement with a study by Gupta et al. [103], it was found that the formation of long-chain hydrocarbons was easier in the catalysts with large pores. The best olefin yield was obtained for the catalyst with a pore size of 49.7 nm [107]. Catalysts with a smaller pore size exhibit confinement effect: small crystallites of the Fe_2_O_3_ phase were formed inside small pores. Their reduction at 400 °C was more difficult, and, in consequence, a smaller amount of active phase was formed [107].

**Figure 14:**
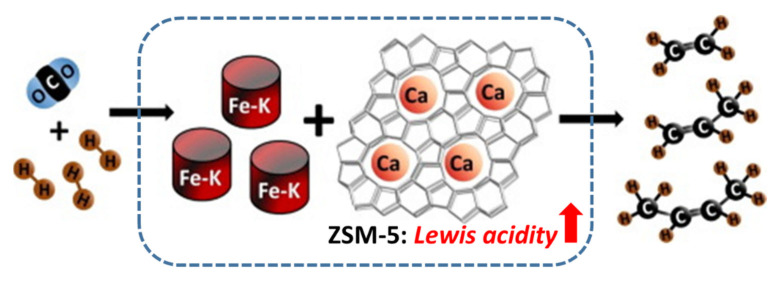
Visualization of the light olefin formation via CO_2_ hydrogenation over K-promoted iron catalyst supported on ZSM-5 zeolite modified with Ca. Adapted from [108] with copyright license from Elsevier (2021).

With the aim to increase selectivity to light olefins, Dokania et al. [108] modified the acidity of the zeolite ZSM-5 by incorporation of Ca by incipient wetness impregnation (Figure 14). This incorporation method led to the reduction in Brønsted acidity and the formation of multiple Lewis acidic species inside the zeolite leading to the enhancement of the light olefins production at the expense of longer chain hydrocarbons. The enhancement of the selectivity to light olefins was explained as due to the creation of surface acetate species and suppression of oligomerization favored by the reduction in the zeolite Brønsted acidity [108].

Methanol formed by CO_2_ hydrogenation can be advantageously used for propylene production using Lurgi’s methanol to propylene technology (MTP) [109]. The MTP process uses methanol produced from syngas for the selective production of propylene in a two-step process: first, methanol is dehydrated to dimethyl ether (DME) over an aluminum oxide catalyst, then DME is transformed over a ZSM-5-based catalyst into a variety of olefins with propylene being the main product. Recently, using this technology, the effect of zeolite modification for enhancement of catalyst lifetime and propylene selectivity was intensively studied [110].

The catalytic propene production by coupling the endothermic propane dehydrogenation reaction with the exothermic reaction of CO_2_ hydrogenation was recently proposed by Liu et al. [111]. In this method, propane was dehydrogenated to propene utilizing the heat generated in CO_2_ hydrogenation. The catalyst used was zeolite KIT-6 with V and Fe species introduced into the structure of this highly ordered mesoporous material. The catalyst exhibited a high dispersion of V and Fe active sites and a large specific surface area. Under the reaction conditions studied (C_3_H_8_/CO_2_/N_2_ = 1:4:5, 580 °C, 0.1 MPa), the propane conversion and propylene yield were 37.8% and 32.9%, respectively [111].

### 5.1. Carbon-Based Catalysts

In general, iron catalysts supported on metal oxides show low activity in the direct hydrogenation of CO_2_ to light olefins (FTO). This is mainly due to the strong metal–support interaction. Compared to metal oxides, carbon-based materials generally exhibit moderate metal–support interaction, higher stability in reducing atmospheres and higher water resistance, which facilitates the formation of the active phase [112]. However, the catalytic behavior of carbon-containing catalysts is highly dependent on their nature, as was demonstrated by Gupta et al. for catalysts possessing the active phases (Fe_3_O_4_, Fe, Fe_5_C_2_) encapsulated within a partially graphitized carbon shell [113]. Compared to the catalyst with amorphous carbon in its shell, their catalyst with the well-graphitized carbon shell showed a lower formation of higher hydrocarbons [113].

There are many recent works on the use of carbon nanotubes [114,115,116,117,118,119,120,121], graphitized carbon [113], honeycomb-structured graphene [122] and N-ordered mesoporous carbon [123,124] to support iron-based catalysts. Nitrogen doping of the carbon support is a common practice because it promotes the reduction in the supported iron oxide particles [114,115,116,117,118,119,120,121]. For example, the effectiveness of functionalization of multi-walled carbon nanotubes (MWCNTs) by oxygen and nitrogen in CO_2_ hydrogenation was investigated by Chew et al. [114]. Both carbon-supported iron catalysts were prepared by dry impregnation method using ferric ammonium citrate as a precursor. The best result in CO_2_ hydrogenation was achieved by nitrogen functionalization of carbon. However, due to the unique characteristics of metal–support–reactant interactions, the catalyst showed high undesired selectivity towards methane and light hydrocarbons [114]. Therefore, it could be concluded that nitrogen-doped MWCNTs are not suitable catalyst support for the production of light olefins via CO_2_ hydrogenation. Similarly, metal-free catalysts such as nitrogen-doped graphene quantum dots (NGQDs) were shown to produce mainly CH_4_ over CO [120], while mesoporous carbon support proved to be effective for CO_2_ hydrogenation to liquid carbons due to the easy entry of heavy products within its pore structure [121].

The effect of the use of honeycomb-structured graphene (HSG) as support was investigated by Wu et al. [122]. The catalytic behavior of the K–Fe/HSG catalysts modified with various amounts of potassium was evaluated in reaction of CO_2_ hydrogenation to light olefins (FTO route) [122]. The FeK_1.5_/HSG catalyst, having optimized K loading, showed a high yield of light olefins (73 μmolCO_2_ g_Fe_ ^−1^ s^−1^) and 59% selectivity. This most optimized catalyst was stable during 120 h on stream. Its excellent catalytic behavior was explained as due to the confinement effect of the porous HSG inhibiting the sintering of the active sites and the promoting effect of K favoring CO_2_ activation and the formation of iron carbide species [122].

**Table 2:** Comparison of promoter effect on catalyst efficiency for the formation of C_2_^=^–C_4_^=^ olefins via CO_2_ hydrogenation ^a^.

Catalyst	T (°C)	P (MPa)	H_2_/CO_2_ Ratio	Conversion (%)	Yield ^c^ C_2_–C_4_=	Ref.
Alkali—Fe carbide	270	1.2	3	25.5	8.5	[67]
5%NaFe	290	1.5	3	34.7	18.8	[66]
K–Fe–Al–O (nanobelts)	300	1	4	48.0	25.0	[125]
K–Fe–Co/Al_2_O_3_	300	1.1	3	31.0	17.9	[51]
In_2_O_3_/ZrO_2_+SAPO-34	400	1.5	3	19.0	16.2	[8]
Fe–Co/K–Al_2_O_3_	320	2	3	49.0	18.1	[107]
Fe_3_O_4_ (microsphers)	350	2	3	43.0	15.7	[78]
10Mn–Fe_3_O_4_ (microspheres)	350	2	3	44.7	18.7	[78]
M–InS/Fe–Co	360	2	3	50.9	11.5	[106]
T–InS/B–FeCo	360	2	3	48.0	21.5	[106]
T–FeCo/B–InS	360	2	3	47.7	16.2	[106]
NiCu/CeO_2_–SAPO-34	375	2	3	15.3	11.7	[88]
13%ZnO–ZrO_2_/MnSAPO-34	380	2	n/a ^b^	21.3	13.1	[126]
ZnO–ZrO_2_/SAPO-34	380	2	3	12.6	10.1	[40]
0.5%NaFe	320	3	3	38.4	24.7	[63]
Mn/Na/Fe	320	3	3	38.6	11.7	[62]
ZnGa_2_O_4_/SAPO-34	370	3	3	13.0	11.2	[102]
MgGa2O4/SAPO-34	370	3	3	8.7	1.0	[102]
ZnGa2O4/SAPO-34	370	3	3	13.0	6.0	[62]
ZnAl2O4/SAPO-34	370	3	3	15.0	6.7	[102]
In–Zr(4:1)/SAPO-34	380	3	3	26.2	19.5	[97]
K–Fe–Co–Zr fibers	400	3	3	42.0	12.6	[58]

^a^ Molar percentage; ^b^ n/a: not available. ^c^ Yield(%) = Selectivity toward C_2_^=^–C_4_^=^ (%) × CO_2_ conversion.

**Figure 15:**
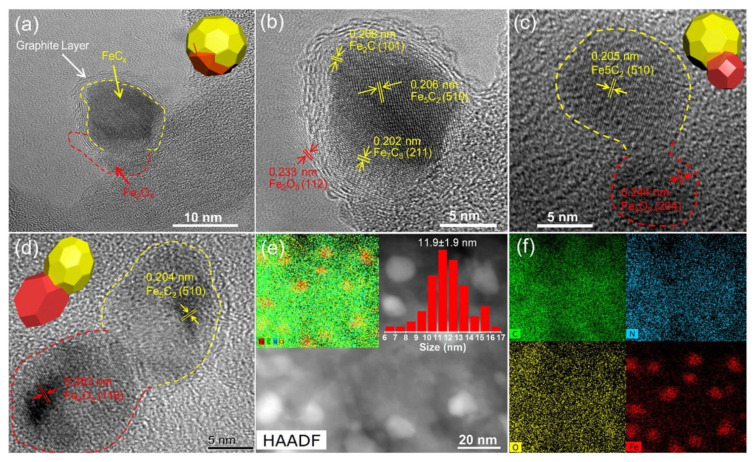
(**a**–**d**) HR-TEM images and (**e**,**f**) HAADF-STEM images and elements mapping of Fe_2_O_3_–FeCx@N-OMC catalyst with active phases confined in N-doped ordered mesoporous carbon. Fe_2_O_3_–FeCx heterojunction shown in (**d**). Reproduced from Ref. [123] with copyright license from Elsevier (2021).

Recently, Zhang et al. [123,124] reported excellent activity and stability results for Fe_2_O_3_–FeCx@N-OMC catalyst with active phases confined in N-doped ordered mesoporous carbon (N-OMC). In these catalysts, the carburization process led to the formation of Fe_5_C_2_, Fe_7_C_3_, Fe_2_C and Fe_3_O_4_–FeC_x_ phases, the latter having heterojunction structure (Figure 15). Catalysts confining hybrid phase showed excellent activity and selectivity towards olefins formation from CO_2_ via the RWGS-FTO route mechanism, in which the Fe_3_O_4_ phase was responsible for catalyzing the formation of CO by the RWGS reaction, while the light olefins were formed by the FTO reaction catalyzed by the neighboring FeC_x_ active sites. The enhancement of the catalyst activity was attributed to the synergy Fe_3_O_4_–FeC_x_ heterojunction. Due to the confinement of the actives inhibiting reoxidation and agglomeration of the active phases, the catalyst showed excellent stability during the time course of CO_2_ hydrogenation to light olefins [123].

### 5.2. Effect of the Catalyst Preparation Method

The most commonly used methods for the preparation of iron catalysts are aqueous precipitation or hydrolysis of Fe^2+^ and/or Fe^3+^ salts [22,23]. However, the physical and chemical properties of the solids prepared by the aqueous precipitation methodology depend on several factors, such as precipitating agent, solution concentration, the temperature of the solution, pH, pretreatment temperature, aging and drying conditions, etc. [23]. Iron-based catalysts are usually prepared by the precipitation method, which involves the precipitation of iron hydroxides and iron oxides from an aqueous solution of Fe precursors. Subsequently, the precipitate is washed, dried and treated in air at high temperature. Finally, the catalyst is activated in H_2_ before the catalytic activity test is performed. Unfortunately, the iron catalyst precursor, which is composed predominantly of magnetite (Fe_3_O_4_), possesses a very low surface area (1–15 m^2^ g^−1^), which explains its low activity. Interestingly, a high surface area Fe–K catalyst containing mixed Fe_3_O_4_/γ-Fe_2_O_3_ phases was prepared by Visconti et al. [127] using the rapid decomposition method of ammonium glycol complexes. The catalysts prepared by this method proved to be more active than the reference catalysts K–α-Fe_2_O_3_ and K–Fe_3_O_4_, which was related to its higher BET surface area and better catalyst carburization.

When monometallic catalysts are prepared, the precipitation of iron ions is simple and fast. However, the situation becomes more complicated when bi- or multi-metallic catalytic precursors need to be synthesized. In this case, the precipitation conditions need to be controlled more carefully in order to obtain a homogeneous distribution of the different components. For instance, pH must be adjusted and controlled using a pH-state or a chemical buffer. Thus, the carbonate/bicarbonate buffer can be used by adding a solution of sodium bicarbonate (NaHCO_3_) and bubbling CO_2_ through the solution in order to keep constant the pH around 8.5 when iron nitrates are employed as precursors [23].

The effect of calcination temperature on the catalytic behavior of Fe–Co/K–Al_2_O_3_ catalysts was investigated by Numpliai et al. [128]. High-temperature calcination was found to cause agglomeration of metal oxide particles, which hinders the reduction in iron oxide species. In addition, the high calcination temperature negatively influences the interaction between Fe_2_O_3_ and other metal oxides. However, the decomposition of KNO_3_ into K_2_O was favored. As a consequence of the enhanced interaction between Fe_2_O_3_ and K_2_O and the formation of the KAlO_2_ phase, the undesired hydrogenation of olefins was inhibited. Contrary to CO_2_ conversion and hydrocarbon selectivity, the olefins to paraffin (O/P) ratio followed a volcano-curve trend as a function of calcination temperature. At elevated calcination temperature, the olefins to paraffin’s (O/P) ratio decreased due to the combined factors of increased particle size, worse metal dispersion and a drastic decrease in BET surface area, which adversely affected CO_2_ conversion and product selectivity [128]. However, contrary as expected, after calcination at the highest temperature (800 °C), the Fe–Mn–K–Ce catalyst exhibited an increase in activity and O/P ratio with respect to the same catalyst calcined at 400 °C [129].

**Figure 16:**
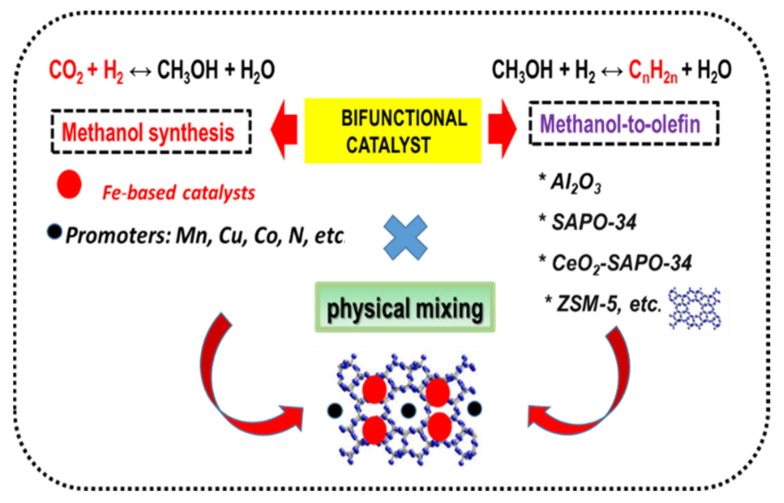
Visualization of the preparation of the hybrid catalyst by physical mixing method. *, supports currently employed.

Unlike catalysts operating via the RWGS-FTS route, catalysts operating via the MTO reaction route are usually prepared by physically mixing the metal components and zeolite (Figure 16). To avoid the considerable cost of zeolite preparation, Tian et al. [129] used the natural sepiolite (palygorskite) to synthesize the CuO–ZnO–Al_2_O_3_/SAPO-34 composite catalyst. The SAPO-34 molecular sieve was prepared using palygorskite as silicon and various templates (diethylamine, triethylamine, morpholine and tetraethylammonium hydroxide), whereas the composite catalysts were prepared by physical mixing of both catalyst components. The best catalyst was prepared by acid-treated palygorskite and tetraethylammonium hydroxide template, which exhibited the highest amount of strong acid sites that are needed for the dehydration of methanol to light olefins [129]. Under the reaction conditions employed (400 °C, 3 MPa, H_2_/CO_2_ volume ratio of 3), the CO_2_ conversion over hybrid CuO–ZnO–Al_2_O_3_/SAPO-34 catalyst was 53.5%, while the selectivity to light olefin of 62.1%. Recently, using ZnO–ZrO_2_/SAPO-34 and In_2_O_3_/ZSM-5 bifunctional catalysts, Gao et al. successfully converted ~20% of CO_2_ to light olefins via MTO mechanisms [7,39].

Zheng et al. synthesized K- and/or Na-promoted FeCoCuAl catalysts by precipitation and impregnation [130]. The best results in terms of activity and selectivity were shown by the FeCoCuAl catalyst prepared by simultaneous modification with K and Na. This is because the simultaneous introduction of Na and K causes the enhancement of the catalyst basicity and electron-rich property, promoting the formation of Fe@Cu and Fe–Co@Cu active sites with Cu^0^ as the crystalline core. These effects were advantageous for H_2_ dissociative adsorption and CO_2_ activation, giving high CO_2_ conversion with hydrogenation. The introduction of Na and/or K led to the formation of Fe and Fe–Co-active phase crystals in the order Na < K < K–Na. The electron-rich Fe@Cu (110) and Fe–Co@Cu (200) crystal phases were claimed to be active centers for subsequent dissociative adsorption of H_2_ and formation of O–C–Fe intermediates after CO adsorption (from RWGS). This was beneficial for the carbon chain growth of C_2_+ hydrocarbons, including olefins and alkanes, and exhibited the highest CO_2_ conversion and C_2_+ selectivity of ~53 mol.% and ~90 mol.%, respectively [130].

The decrease in CO_2_ formed during the direct synthesis of light olefins via the FTO reaction is a major challenge for Fe-based FTO catalysts. Recently, this was successfully achieved with the FeMn@HZSM-5 capsule catalyst with FeMn in the core and HZSM-5 as the shell [96]. Compared with the FeMn catalyst, the capsule catalyst showed much better catalytic behavior with higher selectivity toward light olefins, and the CO_2_ selectivity decreased more than 10%. In addition, the capsule structure of HZSM-5 was also found to suppress the WGS reaction due to the effective diffusion of H_2_O. This improvement of the catalyst behavior towards the production of light olefins by the FTO route is due to the fact that the capsule catalysts are bifunctional, and their catalytic behavior proved to be always better than that of the corresponding catalysts prepared by physical mixing [96].

**Figure 17:**
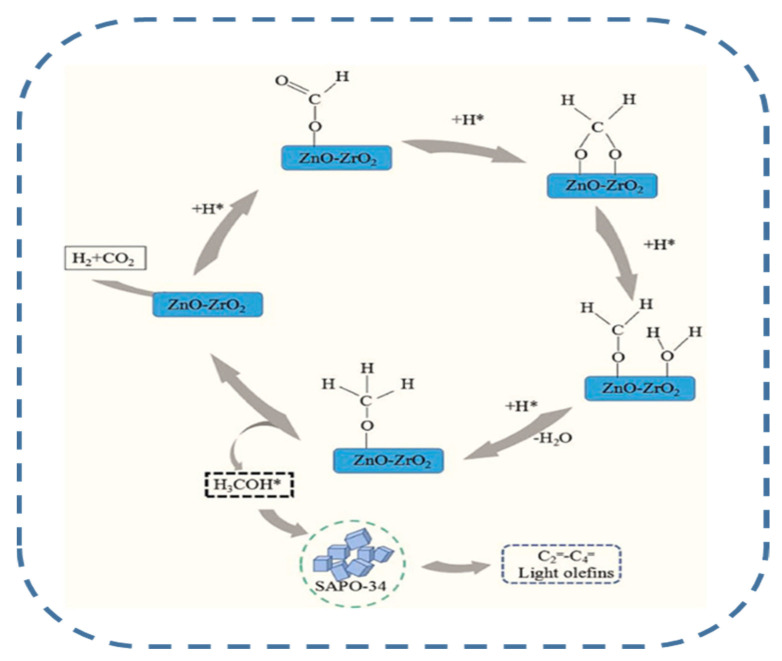
Mechanism of CO_2_ activation on the surface of the 13%ZnO–ZrO_2_ component of the 13%ZnO–ZrO_2_/Mn–SAPO-34 catalyst. Reproduced from [126] with copyright license from Elsevier (2021).

The optimized acidity and shape selectivity effect of the zeolite channels was achieved by physical mixing of Me-SAPO-34 (Me=Mn, Zn, Zr) with ZnO–ZrO_2_ metal oxides [126]. The ZnO–ZrO_2_/Mn–SAPO-34 hybrid catalysts showed the best activity and selectivity towards light olefins: CO_2_ conversion of 21.3% and olefin selectivity of 61.7%. However, in comparison with the other works (Table 2), the yield of olefins was lower. The possible mechanism of CO_2_ activation on ZnO–ZrO_2_ is shown in Figure 17 [126].

**Figure 18:**
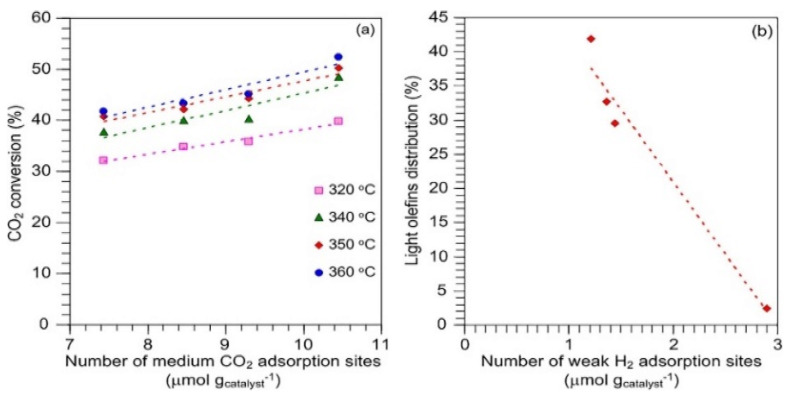
A plot of CO_2_ conversion as a function of medium CO_2_ adsorption sites (**a**) and a plot of light olefins distribution as a function of weak H_2_ adsorption sites (**b**). Reproduced from Ref. [131] with copyright license from Elsevier (2021).

The effect of the catalyst preparation method on the hydrogenation of CO_2_ to light olefins was studied by Witoon et al. [131]. Their Fe–Co–K–Al oxide catalysts were prepared by the precipitation-reduction method using NH_4_OH as the precipitating agent and NaBH_4_ as the reducing agent, and using various synthesis conditions preparation, such as one-pot preparation, without the addition of NH_4_OH, precipitation followed by reduction and doubling the NaBH_4_ content of the precipitation-prepared catalyst. As expected, it was found that the phase, reducibility and adsorption of CO_2_ and H_2_ with the surface of the catalysts changed with different preparation methods. The structure–activity relationships indicated that the CO_2_ conversion was proportional to the amount of medium CO_2_ adsorption sites, while the O/P ratio increased with decreasing amount of weak H_2_ adsorption sites (Figure 18a,b, respectively). In the reaction at 350 °C and 20 bar, the catalyst prepared by precipitation followed by reduction showed a high yield of light olefins (16.6%). This was due to a significant reduction in weak H_2_ adsorption, leading to inhibition of olefin hydrogenation to paraffinic products and a high level of CO_2_ adsorption that provided relatively high CO_2_ conversion. Interestingly, changing the reducing gas from H_2_ to an H_2_+ CO_2_ +N_2_ gas mixture had a negative effect on the reduction in the iron species, as the less active Fe_3_C species were formed instead of the desired active phase Fe_5_C_2_ that was formed using H_2_ for catalyst reduction [131].

Recently, a series of highly efficient ZnCo_x_Fe_2-x_O_4_ spinel catalysts were prepared by Xu et al. via a single-source layered double hydroxide precursor route [132]. Compared to the bimetallic ZnFe_2_O_4_ and ZnCo_2_O_4_ systems, the ternary spinel catalyst ZnCo_0.5_Fe_1.5_O_4_ showed excellent performance in the hydrogenation of CO_2_ to light olefins: at a CO_2_ conversion of 49.6%, the selectivity was 36.1%. During CO_2_ hydrogenation over ternary catalysts, the CoFe alloy phase with electron-rich Fe^0^ atoms was formed. The presence of alloy on the catalyst surface promoted the in situ generations of iron–cobalt carbide, Co_2_C and θ-Fe_3_C phases. In addition to activity enhancement, inhibition of both CO_2_ methanation and secondary hydrogenation of olefins were observed [132].

### 5.3. New Strategies of the Catalyst Preparation

To improve catalyst efficiency in the formation of olefins by CO_2_ hydrogenation, several alternative methods to precipitation, such as electrospinning, microemulsion or microwave irradiation, were recently employed.

The microwave technique is well known in catalysis because of the resulting higher dispersion of nanoparticles, but its use for the preparation of efficient catalysts for the formation of light olefins from CO_2_ is scarce [35,133]. Microwave-assisted precipitation was successfully employed by Zhang et al. for the preparation of Fe–Zn–K [35] and Fe–Zr–Ce–K [134] catalysts. The Fe–Zn–K catalysts were prepared by a microwave-assisted hydrothermal procedure followed by incipient wet impregnation. The catalysts prepared by this method showed higher bifunctional activity towards light olefins and low CO selectivity, but the C_5_+ products were higher, and the probability of chain growth was large. The hydrocarbon distribution was greatly improved on Fe–Zn–K catalysts due to the reduction and surface adsorption behavior of zinc [35]. In order to improve the subsequent CO hydrogenation and CO_2_ adsorption activation, the Fe–Zr–K catalyst was modified with ceria. As expected, varying the ceria content changed the reducibility, surface basicity and surface atomic composition of the catalysts. Therefore, it was concluded that ceria limits the growth of Fe_2_O_3_ crystallites, weakens the interaction between Fe species and zirconia and facilitates the reduction in Fe species [133]. As expected, the ceria-modified catalyst showed increasingly better catalytic performance due to the effect of ceria limiting the growth of Fe_2_O_3_ crystallites, weakening the interaction between Fe species and zirconia and facilitating the reduction in Fe species [133]. Hydrothermal treatment under microwave irradiation is also an effective method for support preparation, as demonstrated with the synthesis of mesocellular silica foam (MCF) from rice husk [134]. The nickel catalyst supported on this material exhibited high structure and catalytic stability, as well as resistance to coke formation in the hydrogenation reaction of CO_2_ to methane [134].

Another interesting method of catalyst preparation is the electrospinning technique. This method was used by Elishav et al. for the synthesis of carburized K/Fe–Al–O catalysts with nanobelts and hollow nanofibers morphology [125]. The catalyst prepared by this technique proved to be efficient for the production of olefins by CO_2_ hydrogenation. The electrospinning technique uses extremely rapid heating during the oxidation of the organic precursors to crystallize the Fe–Al–O spinel phase within the fibers.

Compared to the K/Fe–Al–O spinel powder that produced mainly C_6+_ hydrocarbons, the carburized ceramic nanobelts showed higher CO_2_ conversion (48%) and selectivity to C_2_–C_5_ light olefins (52%). Interestingly, this catalyst appears to be the most efficient for the production of C_2_–C_5_ olefins from CO_2_ when compared to the other catalytic systems recently reported in the literature (Table 2). The activity–structure relationship suggested that the high performance of this catalyst was due to the higher degree of reduction in the iron species and more efficient interaction of the active iron phases with the promoter K. It should be noted that, in addition to the positive effect of K, the insertion of Al inside the Fe_2_O_3_ phase probably modifies the concentration of surface oxygen species and the carburization capacity of the iron species [125].

Kiatphuengporn et al. [135] studied the effects of magnetic field orientation and magnetic flux density on the activity and selectivity of xFe/MCM-41 catalysts with ferro/ferrimagnetic properties. It was found that the application of an external magnetic field to these Fe-based catalysts strongly affected their activity and product selectivity towards light hydrocarbons. Compared to catalysts not exposed to the magnetic field, the xFe/MCM-41 catalysts showed a significant improvement in CO_2_ conversion, while the activation energy was reduced. The improvement in catalytic activities was due to the fact that the magnetic field facilitated the adsorption of reactants and surface reaction on the magnetized xFe/MCM-41 catalysts (ferromagnetism), which led to the decrease in apparent activation energy and the improvement of selectivity towards the formation of C_2_–C_3_ hydrocarbons and methanol [135].

The use of microemulsion technology is an ideal technique for the preparation of materials containing two (or more) metal or oxide phases since the different species are homogeneously mixed within the micelles, resulting in solids with high internal homogeneity and optimal interaction between their constituents (Figure 11) [1]. This methodology was shown to produce more active and selective catalysts compared to catalysts prepared by impregnation and co-precipitation [20,21].

**Figure 19:**
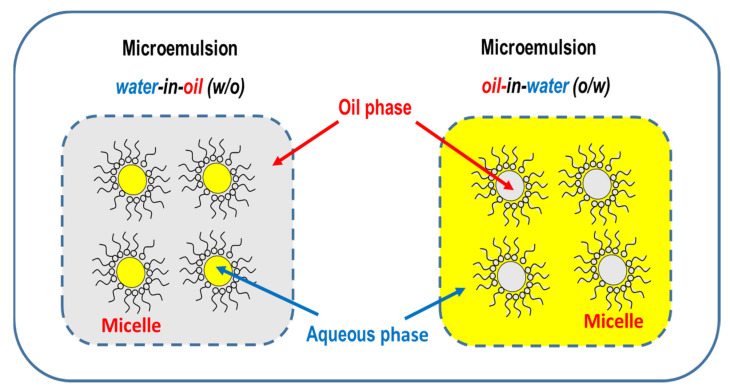
Water-in-oil and oil-in-water microemulsions.

A microemulsion is an optically clear and thermodynamically stable mixture consisting of an organic phase and an aqueous solution stabilized by a surfactant [20,21]. The microemulsion is referred to as water-in-oil (*w/o*) when the aqueous solution is a minor phase or oil-in-water (*o/w*) when the oil phase is a minor phase (Figure 19). It was found that catalysts prepared by this method exhibited better activity in CO hydrogenation than catalysts prepared by impregnation and coprecipitation [20,21]. This microemulsion technique was also successfully employed by Akbari et al. for the preparation of Fe–Co/MgO nanocatalysts [136]. It was found that the air calcined Fe–Co/MgO catalysts tested in CO hydrogenation exhibited higher activity and higher olefin/paraffin ratio than the Ar-calcined ones [136].

## 6. Conclusions

- Despite major advances in catalyst design, the selectivity towards short-chain olefins remains unsatisfactory. In this regard, it is necessary to solve the general problem of olefins desorption. In order to improve olefin formation, it is necessary to modify the functionality of the catalyst by adjusting the strength of the metal–H and metal–C bonds, improving the localization of the active phases, the basicity of the catalyst, the morphology of the support, etc.;
- Iron-based catalysts remain the most widely used catalysts for the production of light olefins via CO_2_ hydrogenation due to their optimal activity/cost ratio. However, although these catalysts were studied for decades, the genesis, the exact nature of the active site and the reaction mechanism under real reaction conditions remain unclear. This is mainly due to the absence of effective in situ characterization techniques and the dynamic changes in the iron phases during the course of the reaction. In this regard, it is necessary to combine experimental work and theoretical study at the molecular level to elucidate the reaction mechanisms in catalysts operating via the FTO and MTO reaction pathways;
- Since the activity and stability of iron catalysts are dramatically increased by incorporating suitable promoters, it is necessary to elucidate the promoter mechanisms to accommodate promoter elements in well-defined and tailored sites to design promoted catalysts capable of producing olefins by the direct CO_2_ hydrogenation route;
- The general problems of catalyst deactivation produced by CO and H_2_O need to be solved. Promising strategies for the improvement of catalyst stability should be the confinement of the active phases within the internal structure of the zeolite or the preparation of catalysts with a core-shell structure. However, the preparation of these catalysts requires very complicated procedures, which increases the cost of the catalyst. In order to decrease the deactivation of the catalyst by H_2_O, tandem catalysts with higher hydrophobicity should be designed;
- Finally, for the direct formation of olefins from CO_2_, new promoted and multifunctional catalysts are expected to be developed, while iron-based catalysts need to be optimized. Among the alternative formulations to iron catalytic systems, those based on In_2_O_3_ have sufficient potential to overcome the limitations observed in conventional iron catalysts. In order to reduce poisoning by the CO product, promising results showed tandem catalysts with metal oxide In_2_O_3_/ZrO_2_ and zeolite SAPO-34 components. Catalysts with a shell-like structure or nanobelts/nanofiber morphology provide good contact between the active phases. However, their complex preparation methods limit their large-scale use for industrial purposes.

## Figures and Tables

**Table 1:** Comparative overview of the features and olefins formation via RWGS + FTS route over Fe and Co–Fe catalysts.

Property	Fe	Co–Fe
Activity	Low CO_2_ conversion	Enhanced CO_2_ conversion
Requirements	Uniform dispersion of active sites and Fe(110) faces for CO_2_ dissociation	A very low Co content Co–Fe_5_C_2_: intimate contact
Selectivity C_2_=–C_4_=	χ-Fe_5_C_2_: higher selectivity than θ-Fe_3_C	Enhanced olefins production
C_5+_ products	Formation of alkanes	long-chain products
CH_4_ formation	High selectivity toward CH_4_	Inhibition of CH_4_ formation
RWGS activity	Active	Co sites: inactive Fe sites: active
Active sites	χ-Fe_5_C_2_: lower hydrogenation ability and chain grown probability than θ-Fe3C:	Co sites: CO dissociation Fe_5_C_2_ sites: hydrogenation and chain growth
Temperature	High (570–630 K)	Low (470–530 K)

## Data Availability

Not applicable.
